# DeGeCI 1.1: a web platform for gene annotation of mitochondrial genomes

**DOI:** 10.1093/bioadv/vbae072

**Published:** 2024-05-13

**Authors:** Lisa Fiedler, Matthias Bernt, Martin Middendorf

**Affiliations:** Department of Computer Science, Leipzig University, Leipzig, Saxony, 04109, Germany; Department of Molecular Systems Biology, Environmental Research—UFZ, Leipzig, Saxony, 04318, Germany; Department of Computer Science, Leipzig University, Leipzig, Saxony, 04109, Germany

## Abstract

**Summary:**

DeGeCI is a command line tool that generates fully automated *de novo* gene predictions from mitochondrial nucleotide sequences by using a reference database of annotated mitogenomes which is represented as a de Bruijn graph. The input genome is mapped to this graph, creating a subgraph, which is then post-processed by a clustering routine. Version 1.1 of DeGeCI offers a web front-end for GUI-based input. It also introduces a new taxonomic filter pipeline that allows the species in the reference database to be restricted to a user-specified taxonomic classification and allows for gene boundary optimization when providing the translation table of the input genome.

**Availability and implementation:**

The web platform is accessible at https://degeci.informatik.uni-leipzig.de. Source code is freely available at https://git.informatik.uni-leipzig.de/lfiedler/degeci.

## 1 Introduction

Mitochondria are small circular organelles that have their own genetic material and can be found in most eukaryotic cells. Mitogenomic research has advanced a wide range of scientific areas, including medicine, forensic genetics, and phylogenetics. The use of massively parallel high-throughput sequencing techniques, which have become accessible in the recent past, is leading to an exponential growth of available mitochondrial sequence data. It is thus getting increasingly difficult to keep up with interpreting, analyzing, and drawing relevant conclusions from these data. Automated and highly efficient pipelines for reliable *de novo* annotation of mitogenomes are hence becoming more and more important. DeGeCI (Fiedler *et al.* 2023) implements such a pipeline by using a de Bruijn graph of pre-curated annotated mitochondrial genomes as reference database, which is mapped to the input sequence and further processed in multiple stages. Here, we present the follow-up version, DeGeCI 1.1, which has three new features that improve the results and make the tool easier to use and more flexible: A taxonomic filter, corrections of gene boundary predictions, and a web front-end.

## 2 Taxonomic filter

In contrast to DeGeCI, DeGeCI 1.1 allows to consider only a subset of the database species of a certain taxonomic classification for the annotation. To this end, DeGeCI 1.1 uses a phylogenetic tree, which stores all phylogenetic relationships between the species contained in the DeGeCI 1.1 reference database. This tree is derived from the NCBI taxonomy database (Schoch *et al.* 2020), which provides a curated classification of a large selection of organisms, accounting for approximately 10% of the known species. The tree is stored in a graph database, where each node represents a certain taxon, which points to the node of its direct ancestor, with the root node representing the origin of life. The nodes are annotated with several attributes, including the taxonomy identifier, scientific taxonomic group name, and common name. If applicable, optional properties, such as synonymous names or labels of mitogenomes in the DeGeCI 1.1 reference database that have the taxonomic classification of the node, are stored.

So far, DeGeCI 1.1 offers two filters. In both cases, it is possible to specify one or multiple taxa. The corresponding nodes can quickly be retrieved from the tree through Elasticsearch indexes, allowing for efficient full-text searches. If multiple taxa are provided, their lowest common ancestor (LCA)—the shared ancestor located farthest from the root—is determined. This is done, by identifying the first intersection of paths from the input nodes to the root. Next, all mitogenome annotations that are contained in the subtree rooted at this LCA (or the single input node) are extracted. The first filter only takes these mitogenomes into account for the annotation, while the second filter disregards them.

Possible use cases for these filters include analyzing which genes have been conserved in the input genome with respect to certain taxonomic groups or studying the impact of specific taxonomic groups on the quality of the results. For example, by setting the filter in a way to use database species that are only distantly related to the input species, only genes that have been conserved over a long period of time will be present in the produced results. If the annotation is subsequently performed on more closely related species, genes that have undergone mutational changes in the recent past start to become visible. By repeating this process, it is therefore possible to study when such mutational events occurred. Moreover, this filter can also be beneficial in terms of annotation time if only closely related database species are used for the annotation (see Section 5).

## 3 Correction of gene boundary predictions

As discussed in Fiedler *et al.* (2023), the DeGeCI protein predictions are often slightly inaccurate at their ends. In DeGeCI 1.1, the genetic code (translation table) of the input genome can optionally be specified. This information is then used to reassess the boundary positions of gene predictions that do not begin or end with appropriate start and/or stop codons. To this end, DeGeCI 1.1 adopts the following simple technique of MITOS2 (Bernt *et al.* 2013). If a start codon is located either upstream or downstream of such a start position, the start position is adjusted accordingly. Stop positions are shifted analogously if stop codons are present in their vicinity. In both cases, the search is constrained to a window of at most 6 nt in each direction, as suggested by Bernt *et al.* (2013). If a stop codon is identified within the investigated region of a start position, the search is restricted to the region downstream of the stop codon. The search is limited accordingly if a start codon is detected in the examined region of a stop position.

To investigate the improvement that can be achieved with the above routine, it was applied to the DeGeCI annotations of the sample set of 100 species used for the evaluation in Fiedler *et al.* (2023). Table 1 demonstrates that this procedure has a noteworthy effect, resulting in 93% of the corrected start and end positions corresponding to start and stop codons, respectively.

**Table 1.** Proportion of start and stop codons out of the total number of DeGeCI 1.1 predictions (precision) with and without boundary correction.

Codon type	Without correction	With correction
start	0.08	0.93
stop	0.14	0.93

## 4 Web server

Although we provide detailed installation instructions and set-up scripts for the DeGeCI 1.1 software, the installation process still requires several steps that must be completed. This, for instance, includes building the Docker image from the provided Docker files and executing the database population scripts. This process also requires some time (several hours), storage space, and a Unix-based operating system. Moreover, the annotation must be run from the command line. This might deter some users from testing DeGeCI 1.1. To this end, we also offer DeGeCI 1.1 as a web application, with the aim of making it accessible to a wider audience. To our knowledge, the only similar web service for mitogenome annotation that is also applicable to a broad spectrum of taxonomic groups is the MITOS2 (Bernt *et al.* 2013) web server. As we have shown in our previous work (Fiedler *et al.* 2023), DeGeCI was already able to generate gene predictions of at least the same quality as MITOS2, while requiring less annotation time when using larger reference databases, and allowing for easier, inexpensive database updates. In DeGeCI 1.1, additionally, a taxonomic filter can be used, which is not offered by MITOS2. Moreover, DeGeCI 1.1 aims to minimize the required knowledge about the input genome and user experience. Therefore, the specification of a genetic code is optional, unlike MITOS2, where it is mandatory. We therefore believe that our web service could be beneficial to many users.

**Figure 1.**
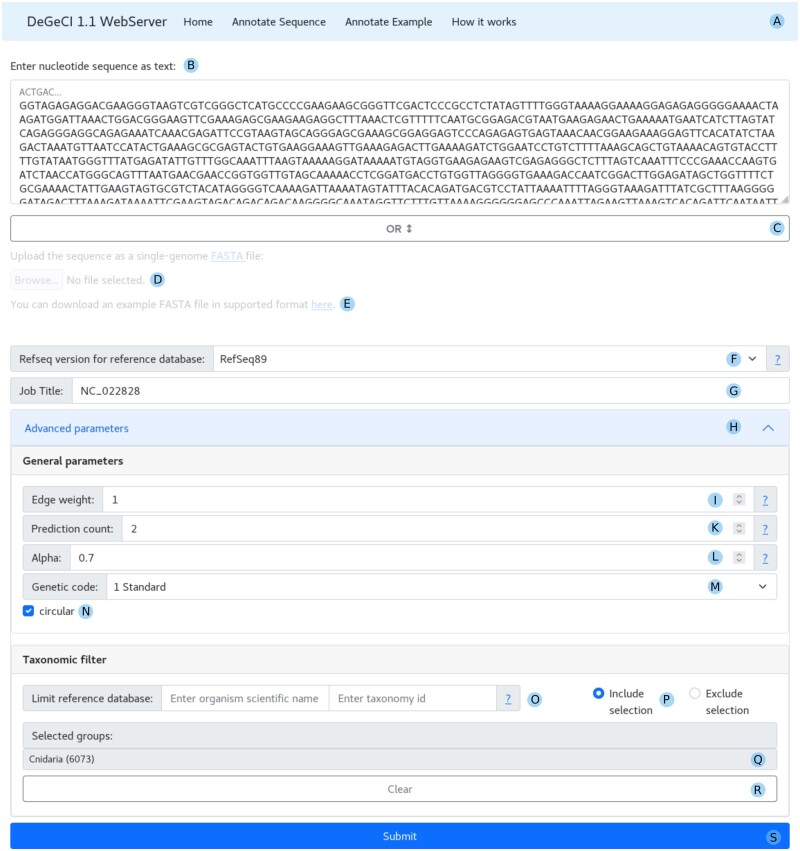
Illustration of the graphical user interface of the DeGeCI 1.1 web application on the example of a coral mitogenome (*Acropora florida*, NC_022828). In this case, the nucleotide sequence is specified as text (B) and Refseq 89 is used as reference database (F). The default settings for edge weight (I), prediction count (K), and α value (L) are not altered. The standard translation table 1 is selected as genetic code (M). The gene boundary correction routine (see Section 3) will thus be applied. Moreover, a taxonomic filter will be used on the reference database. Only species of group Cnidaria will be included (O).

In the following, we present the various features of the DeGeCI 1.1 web service. The website is organized into four different parts, which can be accessed by clicking on the corresponding menu item at the very top of each page (see Fig. 1A). The “Home” page provides an overview and several useful links, including a link to the GitLab repository where the DeGeCI 1.1 software can be downloaded. More detailed information can be found on the “How it works” page. This involves a FAQ section, a description of the algorithmic concepts behind DeGeCI 1.1, how to provide nucleotide sequences for the annotation, how to use the taxonomic filter, as well as the function, default settings, and allowed values of the DeGeCI 1.1 runtime parameters. Moreover, the page explains how to estimate expected annotation times depending on different input conditions and lists average runtimes for input sequences of different taxonomic groups. By visiting the “Annotate Sequence” page, the annotation can be initiated. To get users started, the “Annotate Example” page provides guidance on how to use the various input fields. To achieve this, the page shows an example scenario with already filled-out fields for the mitogenome of a coral species (*Acropora florida*, NC_022828), as illustrated in Fig. 1.

The input sequence can either be pasted into a text area (B) or be uploaded as a FASTA file (D). To toggle between both options, the “OR” button (C) between input fields (B) and (D) must be clicked. By default, the sequence of the example mitogenome is provided as text. However, to see how FASTA files should be formatted, the FASTA file of this sequence can be downloaded from a link (E). This file can then also be used for the file upload. The DeGeCI 1.1 reference database is built from complete mitogenomes of the Refseq data repository (O’Leary *et al.* 2016). Currently, it is possible to choose between two Refseq releases (F), release Refseq 89 (used by default) and the more recent release Refseq 204. It is obligatory to provide a job title (G). This name will be used as sequence identifier in the produced results.

In general, the default parameter settings of DeGeCI 1.1 were found to produce good results for a broad spectrum of different taxonomic lineages. However, adjustments can be made in the advanced parameters menu, which becomes available when clicking on the “Advanced parameters” button (H). One option is to change the edge weight (I). This is the minimum number of database genomes that must contain a (k+1)-mer of the input sequence for the (k+1)-mer to be included in the initial subgraph. Alterations can also be made to the prediction count (K), which is the minimum number of database entries predicting a certain gene annotation for it to be considered, and the α value (L), which determines the highest relative frequency of the relative frequency distribution that must at least be achieved by a cluster to get selected from the dendrogram produced by the DeGeCI 1.1 clustering routine. Each of these input fields has a tooltip, which shows short descriptive texts of their function. In addition, the “?” buttons at the end of each field lead to a section on the “How it works” page, which provides more detailed information.

By default, the input genome is assumed to be circular. Unchecking the check box (N), allows the input to be treated as linear instead. By specifying the genetic code (translation table) of the input genome in the drop-down menu (M), the gene boundary precision correction routine (see Section 3) will be applied to the DeGeCI 1.1 annotations. For the example genome, the standard code is selected, which is the default setting.

To employ the taxonomic filter (see Section 2), either the scientific name of a taxonomic group or its taxonomy ID can be specified in (O). Once the user begins typing, a list of possible completions will appear for convenience. By clicking on an item in the list or pressing ENTER, the entry is added to the list of selected groups (Q), e.g. Cnidaria in the example scenario. Multiple groups can be used for the filter by repeating the above process. Clicking on the “Clear” button (R) removes all selected groups. Radio buttons (P) allow to specify if the filter should be used to include or exclude species from the reference database.

**Figure 2.**
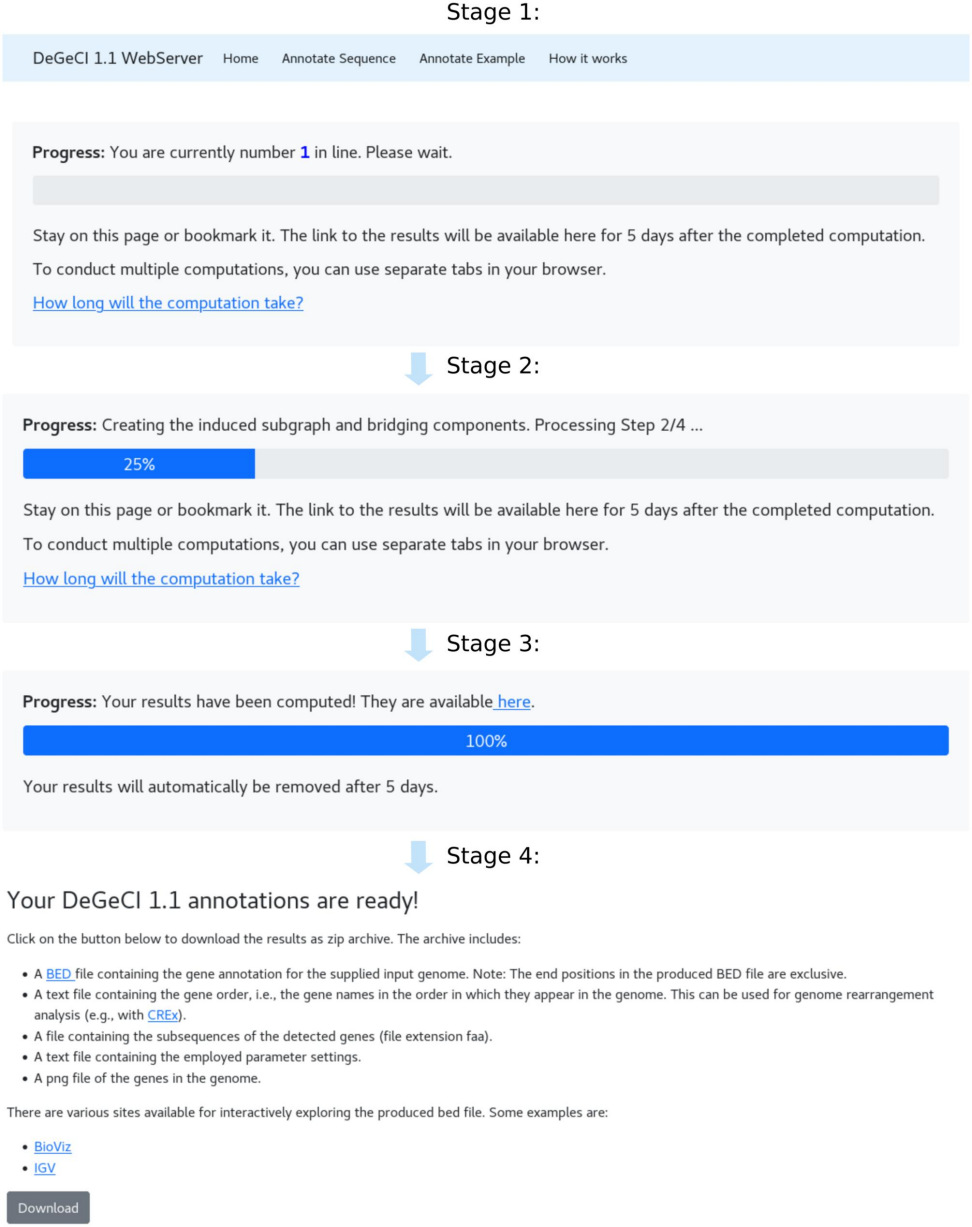
Illustration of the four progress stages of the DeGeCI 1.1 pipeline. Stage 1: The number of pending jobs in the queue is shown in bold font. The computation begins as soon as the number zero is displayed. Stage 2: The computation has begun. A progress bar and a short text above inform about the current status of the computation. The image shows a snapshot of the second computation step. Stage 3: The input genome has been annotated and a link appears. Stage 4: Clicking on the link in Stage 3 leads to the download page, where the produced results can be downloaded as a zip archive.

The job can be submitted once all required input is provided and valid parameter settings are selected. If the specified parameters are invalid, the border of the corresponding fields changes to red and a short message appears, stating what needs to be changed. After job submission, the user is redirected to a new page (see Fig. 2). Initially, the page shows the current number of pending jobs in the queue (Stage 1). Once the number zero appears, the computation begins (Stage 2). A text message and a progress bar inform about the state of the computation. As soon as the job is finished, a link becomes available (Stage 3). Clicking on it redirects to the download page, which also summarizes the produced output files (Stage 4).

## 5 Runtimes on the web server

The annotation times on the DeGeCI 1.1 web server depend on various conditions. In addition to the current server load, this includes the number of queued computations, the length and type of the input sequence, the taxonomic classification of the genome, the choice of runtime parameters, and the taxonomic filter.

Of the runtime parameters, the edge weight has the largest impact. Increasing it results in a greater number of unmatched regions in the initial subgraph, which causes longer bridging times. In general, we have found that selecting the taxonomic filter to exclude closely related species, on average triples the running time. Once again, this is due to large unmatched regions in the subgraph, resulting from the more distinct species used for the annotation. In contrast, choosing the filter in a way including only closely related species, generally results in comparable running times to those without a filter, with a slight tendency towards shorter times. This is because this can reduce the complexity of the subgraph without causing large gaps. However, we emphasize that this can also increase the annotation time. For example, the input sequence might belong to a taxonomic group in which pronounced genome mutations occur at short timescales, again resulting in large unmatched regions.

To provide the user with a rough estimate for concrete times, we have measured average runtimes on the server using a sample of 100 complete mitogenomes with an average length of 16 500 nt, comprising seven major metazoan groups: Actinopterygii (1.9 min), Amphibia (5.60 min), Arthropoda (2.54 min), Mammalia (3.27 min), Sauropsida (5.10 min), Spiralia (4.29 min), and non-bilaterian species (1.54 min). The times were measured using the default parameter settings and no taxonomic filter. They are also listed in the runtime section of the “How it works” page and linked during the progress page of the computation (see Fig. 2).

## 6 Implementation

The taxonomy graph is stored in a JanusGraph graph database with Cassandra storage backend. The web application is implemented in JavaScript using the Node.js runtime environment and the PUGHTML rendering engine.

## 7 Conclusion

This contribution presents DeGeCI 1.1, the follow-up version of DeGeCI. DeGeCI 1.1 enhances DeGeCI by a boundary correction routine to improve the annotation quality, a taxonomic filter to consider only database species of a certain taxonomic classification for the annotation, and a web server front-end to make the software accessible to a wider audience. As DeGeCI, DeGeCI 1.1 is optimized for the annotation of complete mitogenomes. While it is also possible to annotate incomplete mitogenomes, the result quality might suffer in such a case. DeGeCI 1.1 is also not designed to detect misassembled mitochondrial genomes, e.g., in metagenomic samples. However, adapting the taxonomic filter to allow for this task could be an interesting area for future work.

## Data Availability

Source code and installation guides for the DeGeCI 1.1 software are available at https://git.informatik.uni-leipzig.de/lfiedler/degeci. The web platform is accessible at https://degeci.informatik.uni-leipzig.de. The genome sequences of Refseq 89 and Refseq 204 used for the generation of the reference database are available at https://zenodo.org/records/8101631. The taxonomic information of the GenBank taxonomy database is available at ftp://ftp.ncbi.nlm.nih.gov/pub/taxonomy.
